# Crack identification and detection in mine boreholes using deep learning: A case study of hexi coal mine

**DOI:** 10.1371/journal.pone.0353249

**Published:** 2026-11-05

**Authors:** Ruifeng Huang, Wanshun He, Lu Chen, Yanbin Xiang, Wenzhe Li

**Affiliations:** 1 School of Emergency Technology and Management, North China Institute of Science & Technology, Langfang, China; 2 CCTEG Chongqing Research Institute, Chongqing, China; 3 State Key Laboratory for Tunnel Engineering, China University of Mining & Technology, Beijing, China; 4 China National Administration of Coal Geology No.173 Exploration Team, Baoding, China; 5 Sinosteel Shijiazhuang Engineering Design and Research Institute Co., Ltd., Shijiazhuang, China; Islamic Azad University Mashhad Branch, IRAN, ISLAMIC REPUBLIC OF

## Abstract

The internal cracks in deep rock masses are an important factor determining the safety of coal mining. The borehole peeping technology is an important method for obtaining the distribution of coal and rock fractures in deep geological engineering. However, there are often problems such as strong background interference and complex joints in real mines, which bring greater challenges to obtaining the distribution of cracks. Therefore, this study proposes a deep learning-based method for identifying internal cracks, which is used for the intelligent identification and analysis of cracks in drilled rock masses. The experimental verification was carried out based on the real borehole peeping images of the tunnels in Hexi Coal Mine as the data set. By embedding the ResCBAM attention mechanism into the YOLOv8 head network, the reliability and accuracy of identifying internal cracks in rock were further enhanced. The test results show that, the proposed Res-YOLOv8 algorithm can more accurately suppress the interference of complex backgrounds and present the morphological characteristics of rock cracks to compare with the fusion of typical attention mechanisms SE and CBAM with YOLOv8. The ResCBAM-YOLOv8 algorithm demonstrates strong performance in the evaluation indicators of confusion matrix, loss rate, precision, recall and MPA. The proposal of method has improved the efficiency of detecting and identifying cracks in coal and rock masses in real mines, and provided more reliable technical support for the stability evaluation of coal mine roof.

## 1. Introduction

Due to the increasing depletion of shallow resources, the depth of coal mining is constantly increasing. In the deep mining and excavation, fractures, faults and fracture zones of different scales often develop within the rock mass, which are important factors determining the safe mining of coal mines and seriously affect the stability of deep coal and rock [1,2]. The stability of coal and rock mass in the mining area is directly related to the safe production of coal mines. The development degree, distribution pattern and extension range of fractures in the coal and rock mass of the roof are the key geological basis for judging the stability of the surrounding rock of the roadway and formulating scientific support plans [3]. Therefore, the rapid identification of deep coal and rock fissures plays an irreplaceable role in evaluating the stability of coal mine roof.

The methods for identifying rock mass cracks include the traditional line measurement method [4], photogrammetry technology [5], and three-dimensional laser scanning [6], etc. The traditional line measurement method involves reaching the site with tools such as compasses and measuring ropes for manual measurement, which has drawbacks such as low efficiency, high difficulty and low safety factor. However, the photogrammetry methods have high requirements for light and are often difficult to implement in real mines. In addition, three-dimensional laser scanning technology can only measure surface fissures and cannot meet the measurement requirements for the narrow Spaces and dim light underground. Borehole peeping technology [7–9] by drilling holes into the interior of coal and rock masses and collecting high-definition images, it can visually present the development status of fractures, rock layer interfaces, and geological defects within the rock mass, converting hidden geological structures into visual data, and providing precise basic geological support for subsequent geological analysis and engineering decision-making.

However, the borehole peeping technology is affected by the complex geological environment of the coal mining area. The influence of borehole peeping generally has factors such as strong background interference, coal dust obstruction, disordered natural texture of the rock wall, and reflection on the inner wall of the borehole, which are easily confused with the characteristics of cracks. Meanwhile, the scale of cracks targets varies greatly, and the proportion of tiny crack pixels is extremely low, which makes it very easy for small target cracks to be missed. The traditional methods for identifying fractures rely on manual interpretation, which are inefficient and highly subjective. With the introduction of deep learning methods, many scholars have utilized deep learning models for the identification and detection of cracks [10–15]. For example, Zhao et al. used a transformer and a multilevel cross scale weighted feature fusion module to identify the bridge cracks, which have been achieved significant performance improvements in crack detection [16]. Xia et al. connected the digital image correction and a crack tracking algorithm to calculate the stress intensity factor, and the convolutional neural network were proposed to verify the stress intensity factor by inputting rock images [17]. Ma et al. proposed a RESD-CNN algorithm to identify rock damage states, and the model was validated across diverse rock type datasets, achieving an accuracy rate (ACC) of 99.04%, precision of 99.39%, recall of 99.52%, and an F1 score of 99.46% [18]. Si et al. given a identifying coal and rock method based on the convolutional neural network, which introduced dropout, weight regularization, and batch normalization to solve the overfitting problem [19]. However, the existing deep learning detection algorithms generally have problems such as too many iterations, poor adaptability to complex backgrounds, and insufficient detection accuracy for small targets, making it difficult to meet the high-precision and accuracy requirements for crack identification on site.

Therefore, this study proposed an improved YOLOv8 algorithm ResCBAM-YOLOv8 that integrates the ResCBAM attention mechanism. This algorithm use the ResCBAM attention mechanism into the YOLOv8 network to enhance the feature transfer efficiency through residual connection assistance, precisely focuses on fracture targets. Moreover, it precisely focuses on fissure targets and suppresses background interference by combining channel-space dual-dimensional attention. Then, the imaging scanning was conducted using a ZKXG30-type mine borehole inspection device, based on the borehole images from the 3303 working face at Hexi Coal Mine. The device can effectively identify borehole diameters ranging from 50 to 200 mm, and a dedicated fracture dataset was constructed for model validation. The experimental results show that the train loss, Precision, Recall and Mean Pixel Accuracy (MPA) of the proposed ResCBAM-YOLOv8 model are reach 94.1%, 93.5% and 95.2% respectively. To compare with the fusion of typical attention mechanisms SE and CBAM, there are significant improvements. The results obtained using the confusion matrix prove most effective, successfully meeting the requirements for fracture detection in mining boreholes. The proposed model provides reliable technical support for evaluating roof stability.

## 2. Borehole peeping test

### 2.1 Project overview

This study conducts borehole peeping work based on the transportation roadway of the 3303 working face of Shanxi Hexi Coal Mine as the engineering background. The main mining coal seam of the mine is the No. 3 coal seam. According to the lithological distribution of the roof of the No. 3 coal seam, it can be known that the average thickness of the No. 3 coal seam is 1.83 m, which belongs to a medium-thick coal seam. The average dip angle is 6°, and it belongs to a nearly horizontal coal seam. The direct top of the 3303 working face is sandy mudstone with a thickness of 2.24 m, the base top is fine sandstone with a thickness of 3.56 m, the direct bottom is mudstone with a thickness of 1.19 m, and the base bottom is sandy mudstone with a thickness of 7.99 m. The comprehensive bar chart of the top and bottom plates of the 3303 working face is shown in Fig 1.

**Fig 1:**
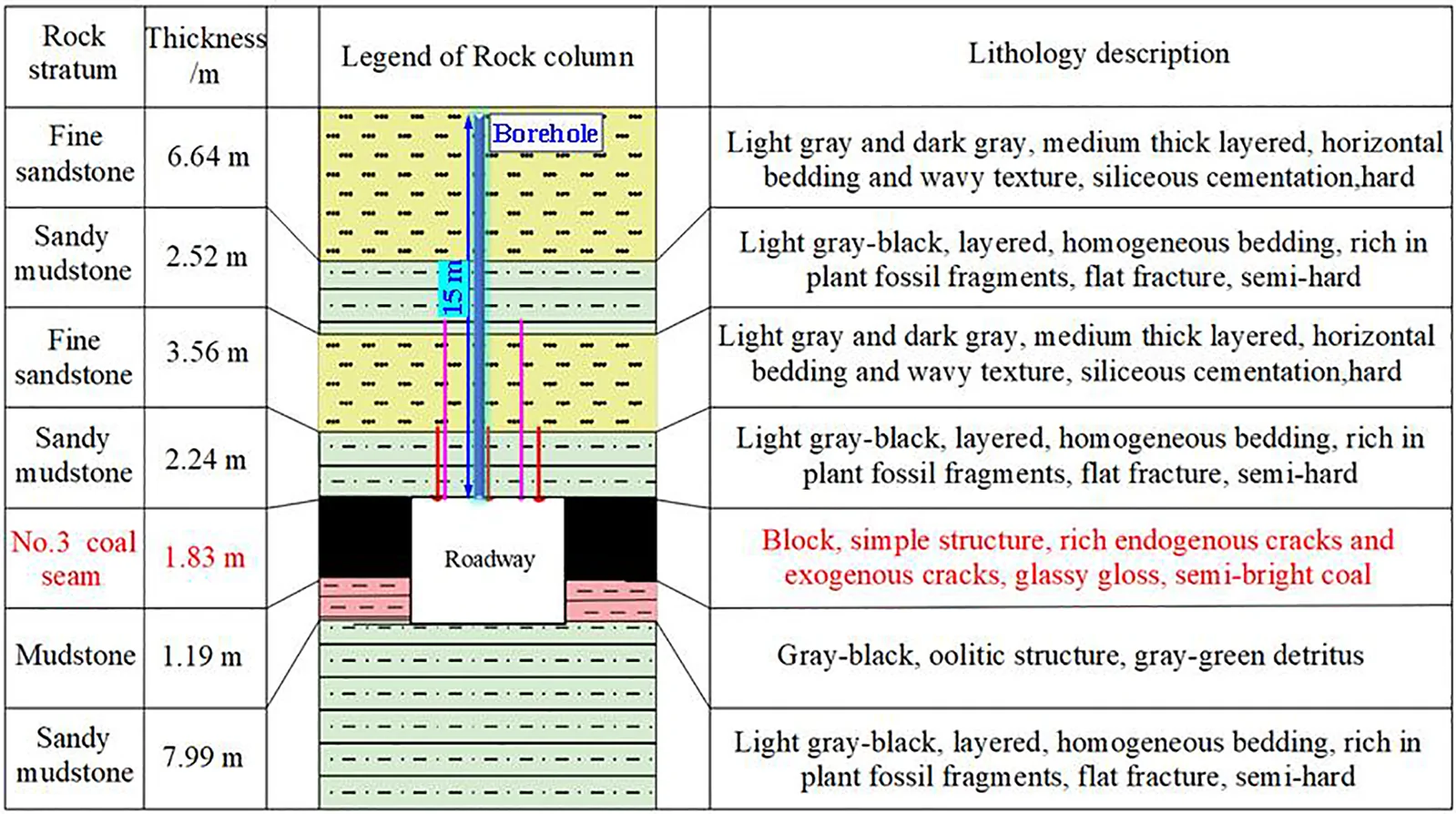
Comprehensive bar chart of the top and bottom plates of the 3303 working face.

### 2.2 Test equipment

To visually obtain the distribution characteristics of cracks and crack zones inside the coal and rock in the complex underground environment of the roadways, a mine drilling imaging trajectory detection device was adopted for detection, as shown in Fig 2. The ZKXG30 mine borehole peeping mainly consists of two parts, namely the main unit of the detection device, the probe, the depth encoder of the mine borehole imaging trajectory detection device and other main components, as well as video transmission cables, signal cables and push rods and other accessories. Among them, the depth sounder is used to record the depth to which the probe travels in the borehole. The probe of the mining drilling imaging trajectory detection device is equipped with an LED white light-emitting diode and a camera to capture images of the hole wall. It is also equipped with a high-performance three-dimensional electronic compass to measure the azimuth and inclination angles of the borehole at the probe’s location. The video signal, control signal and compass digital signal inside the probe are transmitted to the host through the intrinsically safe communication cable for mining. The endoscopic scanning probe has an outer diameter of 40 mm, which can inspect borehole diameters ranging from a minimum of 50 mm to a maximum of 200 mm. The distance is recorded by an internal depth encoder in the push cable, with a measurement accuracy of ±10 mm. The device integrates sensors capable of collecting trajectory parameters such as borehole inclination and azimuth at different depths, enabling evaluation of borehole straightness. The host receives the probe signal and the depth pulse signal of the depth sounders, calculates the depth position of the probe, and processes the video signal such as image recording, matching and splicing. The video recording device can record the entire hole or a part of it. The video recording and image matching are carried out simultaneously. As the probe keeps advancing into the hole, the entire hole wall will automatically match and splice to form a complete planar development diagram. Generally speaking, the mining borehole imaging trajectory detection device integrates functions such as borehole photography, peeping, video recording, imaging, and trajectory measurement. In one test, it can complete the workload of the previous four tests. At the same time, it can obtain dynamic video recording of the borehole, local high-definition photos, full borehole wall layout plans, and the spatial trajectory of the borehole, which is highly efficient and fast.

**Fig 2:**
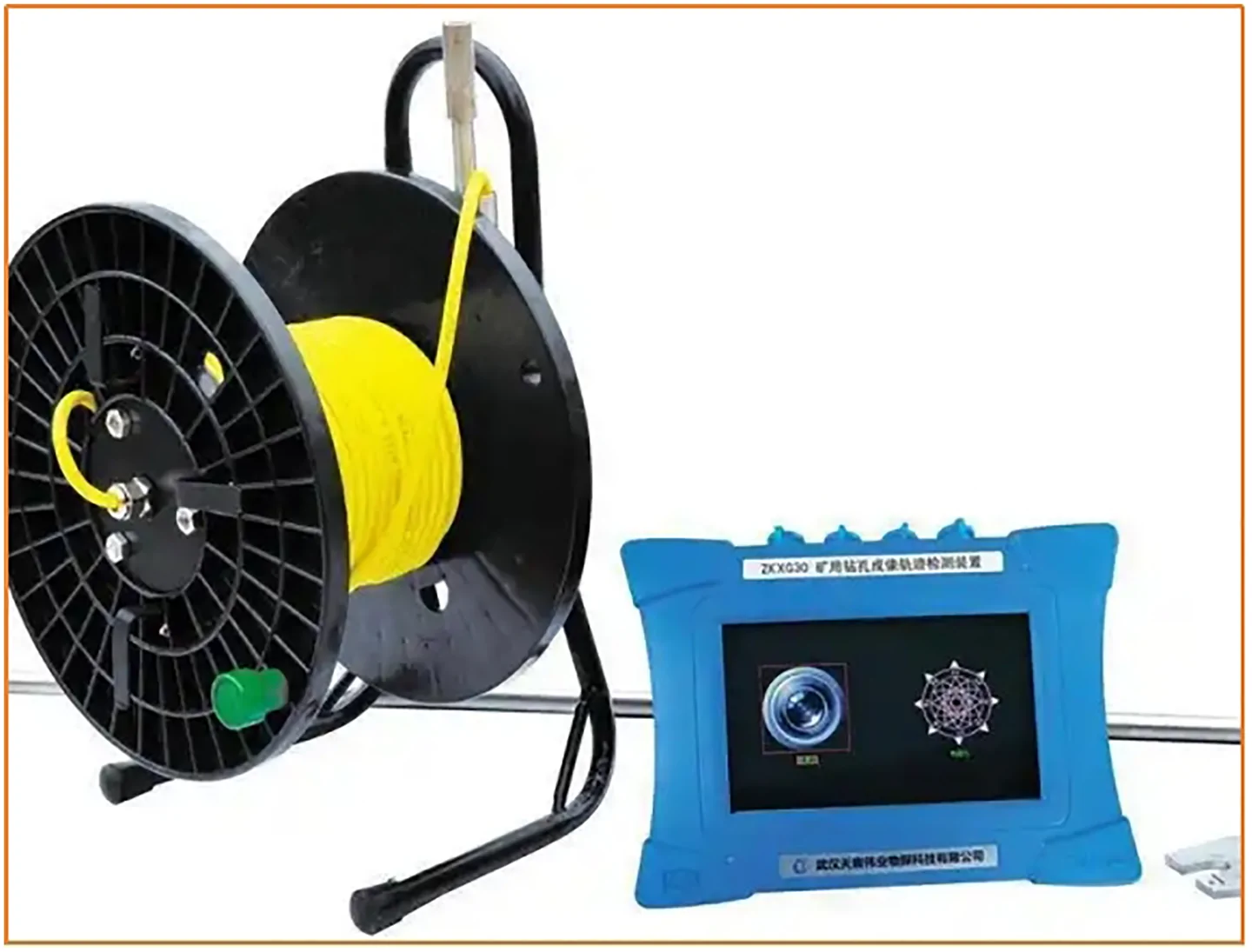
ZKXG30 Mining borehole peeping.

### 2.3 Test procedure

In order to obtain the distribution characteristics of cracks and cracks within the mine borehole peeping, the ZKXG30 mine borehole peeping was used to conduct logging analysis on two different geological boreholes on the upper roof of the roadway in the 3303 working face of a Hexi Coal Mine, and the high-resolution videos of the boreholes were obtained. Among them, the high-resolution video can obtain the features inside the borehole. The specific testing process is as follows:

(1) Determine the measurement points: It is required that the measurement points avoid dense tunnel groups and large structural zones during the layout process. The water, electricity, transportation and ventilation systems near the measurement points should be complete.
(2) Drilling: After the measurement points are determined, the vertical vertical drilling holes are drilled on the roof of the tunnel. The diameter of the drilling holes is 56 mm. The depth of the holes is controlled at 20–25 m according to the roof conditions and the weight of the equipment itself.
(3) Peeping: After the drilling is completed, the mine borehole peeping is used to conduct detailed observations from the hole opening to the bottom of the hole, recording the distribution characteristics of joints, cracks and delamination inside the roof rock layer.

## 3. Construction of crack identification model

### 3.1 Principle of the YOLOv8 Algorithm

The cracks and cracks of coal and rock are often located in the dim underground environment. Due to the uneven lighting and complex textures of background, it is difficult to identify the cracks quickly and accurately. There is an urgent need to construct an optimized algorithm model. YOLOv8 [20,21] is one of the most superior algorithms for object detection, especially in crack identification. The core functions of the YOLOv8 model are object detection and instance segmentation. The object detection mainly uses bounding box annotation technology to identify the category and position information of objects in the image, and simultaneously calculate the probability values of each object category. However, the instance segmentation focuses on classifying individual pixels and generating segmentation masks, which define different categories of objects by using different colors along the contour.

Furthermore, the YOLOv8 model balances the relationship among depth, width and image resolution, thereby significantly enhancing the feature extraction capability and reducing the interference effect. In terms of structural form, YOLOv8 is a new Backbone network, a new Ancher-Free detection Head and a new loss function innovated by the Ultralytics team based on the YOLOv5 network model. The core structure consists of the backbone network and the prediction output part Head, as shown in Fig 3. As can be seen from that, the CSPDarknet structure of the Backbone network are adopted for deep feature extraction of input images, and its core components include the ConvModule module, C2f module, and SPPF module, etc. Among them, the ConvModule module includes two-dimensional convolution, batch normalization, and activation function operations. Its working principle is to systematically slide the convolution kernel on the input image matrix and then perform the convolution operation to generate the feature map. During the training process, by adjusting the weight parameters of the convolution kernel, its ability to learn effective feature representations can be enhanced. The C2f module can ensure the lightweight of the network structure while having better feature extraction capabilities. By achieving the fusion of features at different stages, the model can better utilize features at different levels, enhancing the depth and accuracy of feature extraction. The SPPF module fuses spatial feature information of different scales through the Concat module, enhancing the network’s detection capability for multi-scale targets. The Head prediction output section contains three detection heads, which are used for detection tasks of targets of different sizes. Meanwhile, each detection head has two independent branches, which respectively predict the category of the target object and the coordinates of the bounding box. This operation mode enables each branch to focus on its own tasks, which helps to improve the accuracy and efficiency of target detection results. At the same time, the Anchor-Free frameless structure was used to simplify the model design and further improve the accuracy and speed of the detection network.

**Fig 3:**
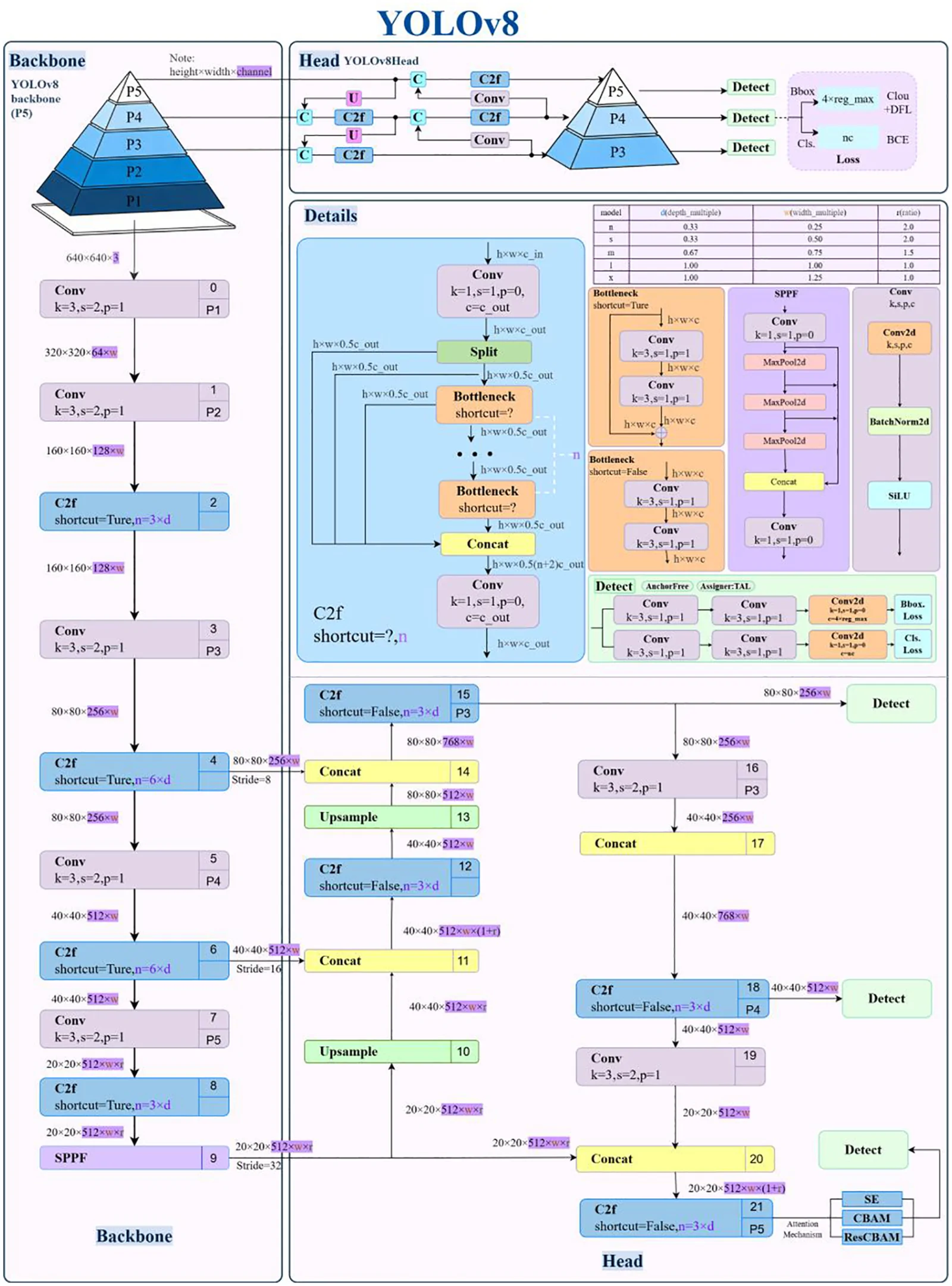
The structure diagram of the YOLOv8 optimization model.

### 3.2 ResCBAM attention mechanism

Due to the advantages of Anchor-Free paradigm, efficient feature extraction module and lightweight design, the YOLOv8 can demonstrate an excellent balance of target detection between accuracy and speed in complex scene. However, the feature extraction processes of the backbone network and neck network are lack targeted enhancement for key areas, making it difficult to adapt to the characteristics of low signal-to-noise ratio and dense targets of borehole peeping images. The attention mechanism [22,23] is a resource allocation mechanism that simulates the human brain’s attention. Its core idea is to reasonably change the attention to information, ignore irrelevant information and magnify the required information. The attention mechanism has become an effective means to improve the performance of target detection in complex scenes by strengthening useful features and suppressing redundant information. The attention mechanisms are divided into channel attention and spatial attention. Firstly, the channel attention mechanism can enhance the feature expression of each channel, and its working principle [24] is as follows: (1)The input feature map is processed through two parallel max-pooling layers and average-pooling layers. The processed results are fed into a shared fully connected layer to learn the attention weights of each channel. (2) These weights are multiplied by each channel of the original feature map to obtain the channel feature map after attention weighting. Secondly, the spatial attention mechanism aims to emphasize the importance of different positions in the image, and its working principle [25] is described as follows: (1) For the input feature map, max-pooling and average-pooling operations are performed. Subsequently, the two resulting feature maps are concatenated, and a convolution layer is used to process the concatenated feature map to generate spatial attention weights. (2) The obtained spatial attention weights are applied to the original feature map, thereby weighting the features at each spatial position. The channel attention mechanism and spatial attention mechanism are as shown in Eq. (1) and (2) as follows:


$$\begin{array}{c} M_{c}(F)=\sigma \left(W_{\text{1}}\left(W_{\text{0}}\left(F_{avg}\right)\right)\right)+W_{\text{1}}\left(W_{\text{0}}\left(F_{max}\right)\right) \end{array}$$ (1)

$$\begin{array}{c} M_{s}(F)=\sigma \left(f^{\text{3}\times \text{3}}\left(\left[F_{avg};F_{max}\right]\right)\right) \end{array}$$ (2)

Where *F* represents the input feature map, $F_{avg}$ and $F_{max}$ are the results after performing average pooling and maximum pooling of channel dimensions on the feature map *F*, respectively. $W_{\text{0}}$ and $W_{\text{1}}$ are the weight matrices of the fully connected layers of the dimensionally reduced layer and the dimensionally increased layer, respectively. σ is the Sigmoid activation function, which can normalizes the results to the interval [0,1]. $M_{c}(F)$ is the final obtained channel attention map, which is used to assign weights to different channels of the feature map. $f^{\text{3}\times \text{3}}$ is a 3 × 3 convolution operation, and $\left[F_{avg};F_{max}\right]$ concatenates two single-channel feature maps along the channel dimension. $M_{s}(F)$ is the final spatial attention map, which is used to assign weights to different spatial regions of the feature maps.

In order to obtain the ${\text{F}}^{\prime }$, the channel attention weight ${\text{M}}_{\text{c}}(\text{F})$ and input feature map are multiplied. Then, the final feature map ${\text{F}}^{\text{'}\text{'}}$ is calculated as follows:


$$\begin{array}{c} F^{\prime }=M_{c}(F)⨂F \end{array}$$ (3)

$$\begin{array}{c} F^{\text{'}\text{'}}=M_{s}(F)⨂F^{\prime } \end{array}$$ (4)

The standard CBAM (Convolutional Block Attention Module) [26] is adjusts the weights of feature maps through channel and spatial attention, which may lead to excessive attenuation of key original features. Especially in deep networks, the layer-wise weight adjustment can accumulate information loss. Moreover, the stacking of standard CBAM modules increases the network depth, resulting in gradient vanishing during backpropagation and thus affecting the training performance of deep networks. The standard CBAM module directly outputs the attention-weighted re-calibrated features. In the borehole inspection images, due to the interference of coal dust and lighting, the fine cracks are weakly responsive features and are prone to be restricted by the attention weights.

Residual Convolutional Block Attention Module (ResCBAM) [27] is an enhanced attention mechanism that integrates Residual connections and CBAM. The core design objective is to alleviate the vanishing gradient problem in deep network training through residual connection based on the attention weighting of the channel dimension and spatial dimension of the feature map by CBAM, while strengthening the transmission and extraction of key features, ultimately improving the recognition accuracy and anti-interference ability of the model for the target area. This study is combines the Channel attention module (CAM) and the Spartial attention module (SAM) and the ResCBAM attention module is obtained through the Residual connection (RC). The core idea of residual connection is to add direct connections between one or more layers of the network so that the network can learn the residuals between the input and output. This approach helps the model to reflux gradients more effectively during the training process, thereby alleviating the problem of vanishing gradients in deep networks. Compared with the standard CBAM module and the cross-attention mechanism module, on one hand, the ResCBAM module retains the underlying original fracture information through the residual branch, while inheriting the channel-spatial joint feature enhancement ability of CBAM. It reduces the information loss caused by the attention module and is more suitable for the detection task of large-scale fracture scales and numerous weak fracture targets. On the other hand, ResCBAM-YOLOV8 has lower computational cost. The global feature interaction of cross-attention is prone to amplify background noises such as coal dust and lighting, and has limited ability to model local tiny fractures. ResCBAM protects the weak fracture features through residual connections, and has better training stability on small-scale borehole image datasets. It can balance the enhancement of multi-scale fracture features and noise suppression, and is more suitable for the requirements of borehole fracture detection tasks. Therefore, the ResCBAM attention module is overcomes the limitations of traditional convolutional neural networks when dealing with information of different scales, shapes, and directions, and alleviates the problem of vanishing model gradients. The structure is shown in Fig 4.

**Fig 4:**
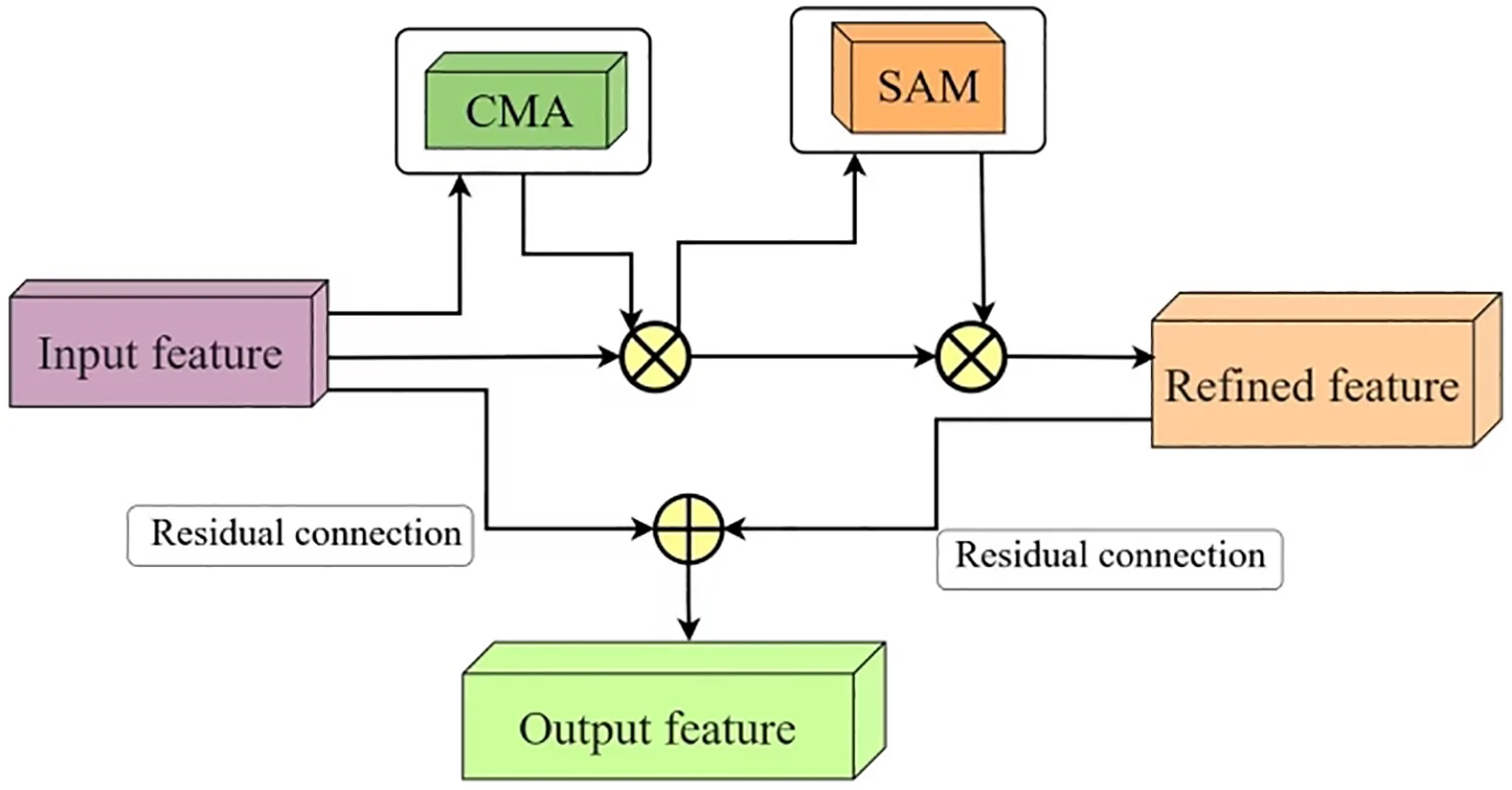
The structure diagram of ResCBAM attention mechanism.

### 3.3 A new model of borehole peeping crack segmentation

In the process of object detection, the traditional YOLOv8 model is fails to fully leverage the crucial feature map information for enhanced feature extraction. Additionally, it is unable to effectively detection the region of interest (ROI) from the background for focused learning, resulting in issues such as low segmentation accuracy and discontinuity at object edges. The ResCBAM attention mechanism is integrates residual connections with a channel-spatial dual attention mechanism, which not only enhances feature representation capabilities but also avoids the problem of gradient vanishing. Therefore, to address the issues of unclear recognition of object details and the inability to focus on learning the ROI during the sampling process in the YOLOv8 model, our study is incorporates the ResCBAM attention mechanism into the YOLOv8 algorithm, constructing an improved model suitable for crack detection in borehole peeping, thereby enhancing the model’s learning ability for cracks. The ResCBAM attention mechanism is added after each sampling in the YOLOv8 model to extract more semantic information. Moreover, to capture tiny cracks in results of mine borehole peeping, our study adds ResCBAM modules after each of the four C2f modules to enhance the capture of key features and suppress interfering information, enabling accurate and rapid identification of cracks in borehole peeping. There are differences between the clay-filled cracks in the borehole observation images and the clearly defined open cracks. The interior of the cracks is filled with clay-rich substances, resulting in a reduced contrast in grayscale with the surrounding coal rock matrix. However, the clay-filled cracks still possess the continuous strip-like morphological feature, which can be distinguished from irregular interferences such as coal dust spots and stains. The proposed ResCBAM-YOLOv8 model mainly relies on this continuous linear morphological feature to identify the clay-filled cracks. Although the gray level difference caused by the clay filling is weakened, the ResCBAM module can still enhance the strip feature response of the filled cracks and suppress the interference of the clay texture, completing the crack detection. These improvements enhance the model’s accuracy and robustness when processing images of complex coal-rock combinations, providing more robust technical support for subsequent coal-rock analysis and applications.

## 4. Experimental analysis of crack segmentation in the new model

### 4.1 Data preprocessing

Due to the insufficient light underground, the color of the coal and rock is relatively dark, and the morphology of its cracks is complex and diverse. To identify the cracks, it is necessary to accurately distinguish the characteristics from the background texture. Moreover, the actual morphology, occurrence and texture features of coal and rock cracks are not complete enough. It is impossible to quickly and accurately identify the cracks, especially the tiny ones, just by observing them with the naked eye. Therefore, this study takes the 3303 working face of Hexi Coal Mine as an example and uses a mine borehole peephole for monitoring. The video results collected inside the borehole are recorded and converted into images for further processing, in order to establish an automatic, accurate and rapid coal and rock crack identification model. Firstly, all image samples are cropped at a resolution of 720 × 480. The original image resolution of probe is not uniform. In this study, the ResCBAM-YOLOv8 model, when inputting image data, will scale all samples to a resolution of 640 × 640 before feeding them into the YOLOv8 network. The scaling process uses bilinear interpolation. Meanwhile, within the training set, multi-scale scaling enhancement is retained. During training, the input size is randomly adjusted within the range of 480–800 pixels to enhance the model’s adaptability to resolution changes brought about by different imaging distances. Since the image samples are extracted from the endoscopic videos of the boreholes, the similarity between the original consecutive video frames is very high. If the training set and test set are randomly divided directly, it is likely to cause data leakage, resulting in an overestimation of the model’s detection performance. Therefore, during the dataset construction process, a large number of highly similar and repetitive images in the consecutive frames are eliminated, and samples with significant differences in crack features are selected for model training and testing. The internal camera images of the boreholes are affected by the on-site conditions, presenting problems such as uneven illumination, contamination and pollution caused by coal dust, and inconsistent resolution due to changes in the shooting distance. To reduce the impact of these disturbances on the crack detection results, the original images are preprocessed before being fed into the ResCBAM-YOLOv8 model for training. The images are mitigated for image brightness imbalance caused by fluctuations in light intensity through brightness enhancement, contrast adjustment and histogram equalization. Gaussian filtering is adopted for noise reduction to suppress the particle noise generated by coal dust and alleviate the blocking effect of coal dust coverage on the crack targets, as shown in Fig 5. By preprocessing the internal camera images of the boreholes, the features such as crack textures and edges in the images are highlighted, thereby enhancing the model’s ability to recognize complex working condition borehole images.

**Fig 5:**
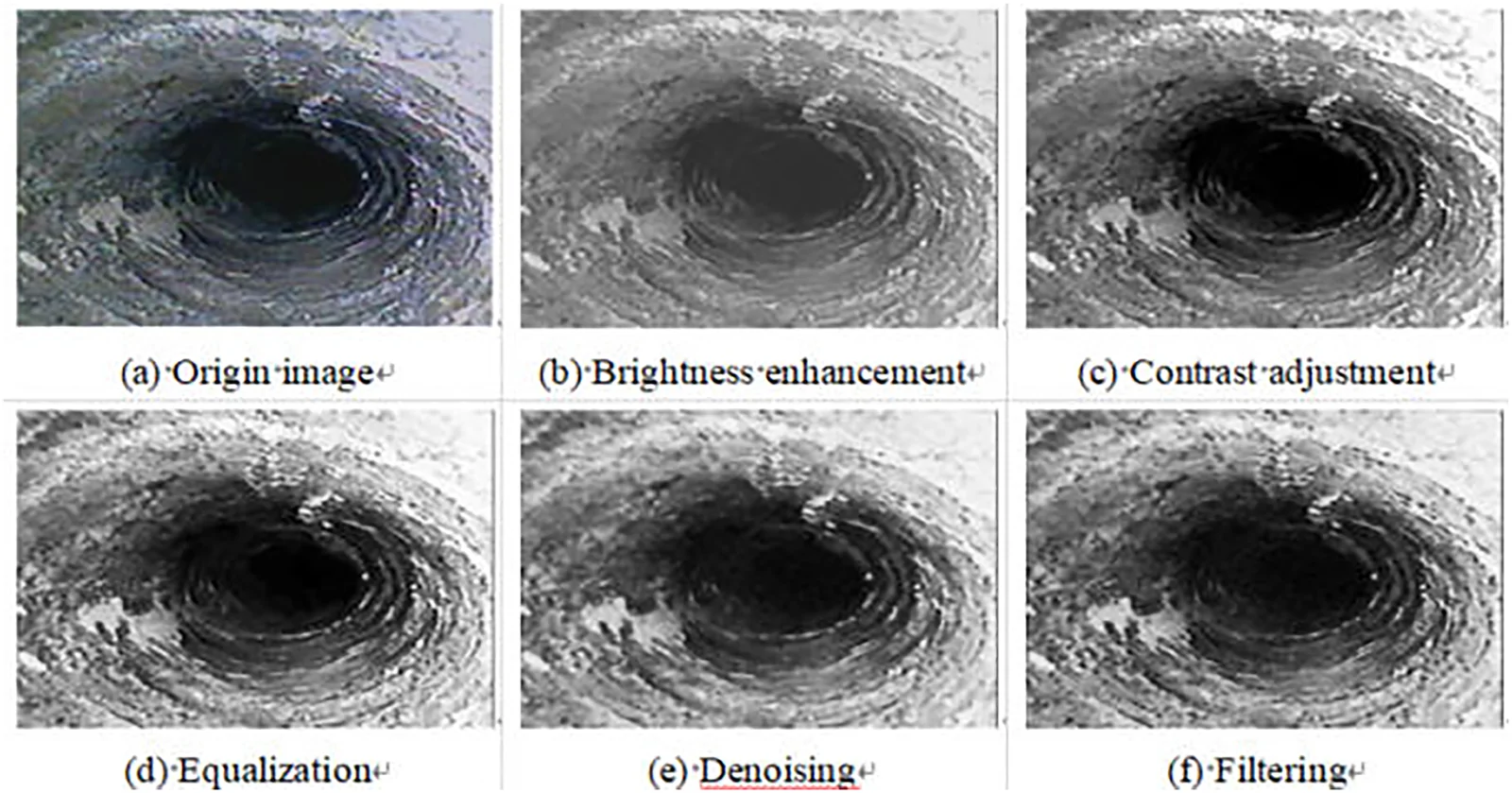
The preprocessing results of crack images observed through mining borehole peeping.

To reduce the risk of overfitting, a combined data augmentation strategy is adopted during the training stage, including random horizontal flipping, random rotation, and random scaling. Since the images are obtained from different regions, the diversity of samples is increased, which can display borehole cracks and cracks of different orientations, sizes and shapes, thereby enhancing the learning ability of the model. Meanwhile, the software Labelme was used to mark and number the irregularly shaped cracks and cracks in the mine borehole images point by point, among which the cracks and cracks were marked as Crack. After image screening and preprocessing, a total of 1667 images with clear and representative cracks were finally selected to form the borehole peeping data set. The data set was randomly divided into a training set and a test set at a ratio of 7:3, that is, 1167 images constituted the training set and 500 images constituted the test set. Fig 6 shows some of the labeled samples used for model training.

**Fig 6:**
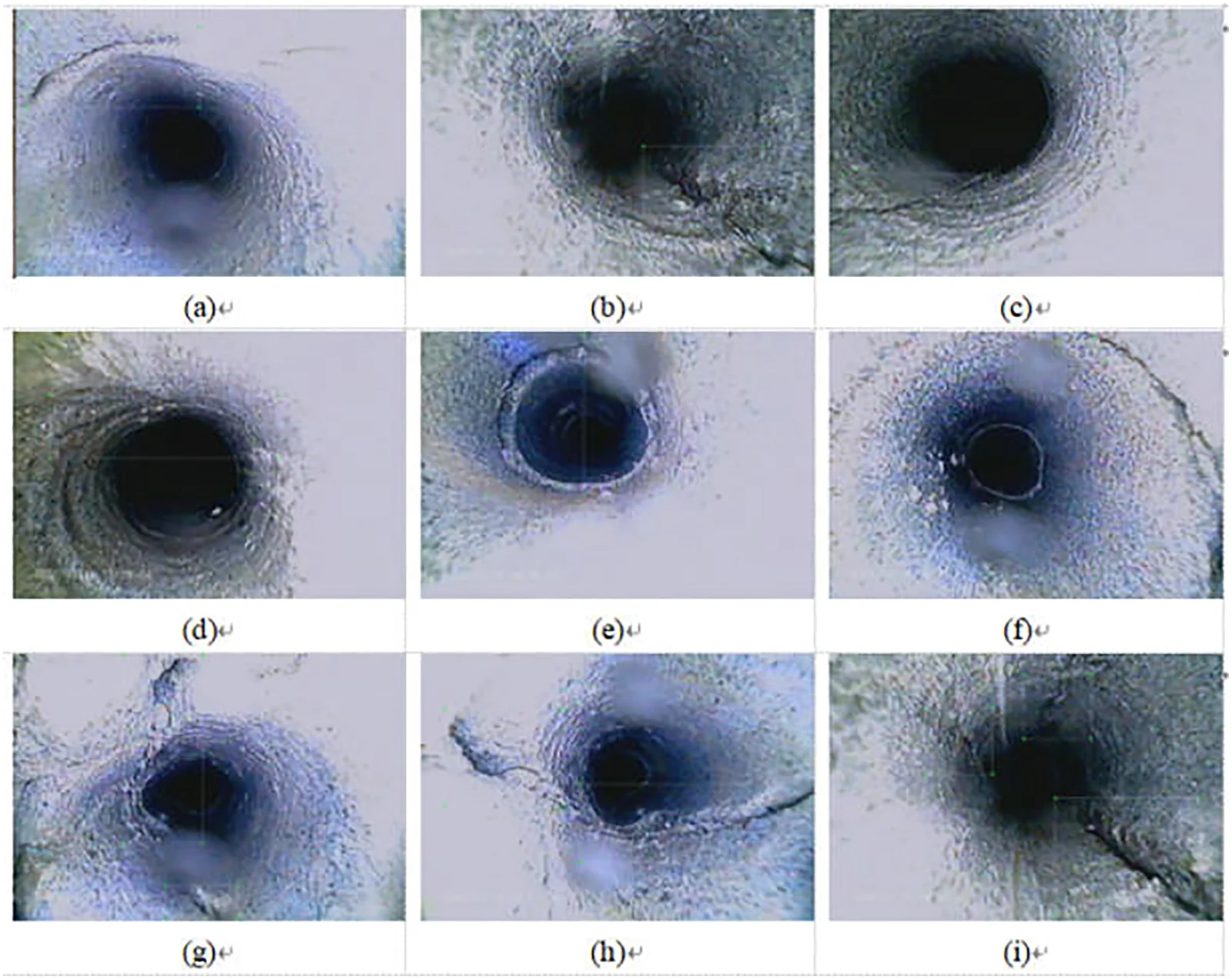
The partial visualization sample slit image marking map.

### 4.2 Parameter configuration

For the task of borehole peeping crack identification, the model training and validation were carried out on a deep learning workstation. The device adopted the Windows 10 operating system and the Intel(R) Core(TM) i7-10700 CPU @ 2.90GHz processor, the 64GB memory and NVIDIA GeForce RTX 3070 graphics card. A deep learning network model is built using Python3.6 based on the Pytorch1.10.2 deep learning framework. During model training, the SGD is adopted as the model loss function optimizer, which can adjust the learning rate of each parameter in the training process, enabling the model to converge to the optimal solution more quickly. To set the learning rate to 0.01 can ensure that the model does not experience sharp fluctuations during the learning process, thereby reducing oscillations near the optimal solution. To set the momentum to 0.9 to ensure the continuity and smoothness of the model when updating parameters. To set the batch number of images processed in the network to 4 and the epoch number of model iterations to 200 can ensure that the model has sufficient opportunities to learn from the training data multiple times, thereby gradually converging to a better state.

### 4.3 Analysis of the test results using the new model

The extraction of borehole peeping cracks in real working conditions is rather difficult. This is generally due to sample imbalance, different crack scales, the presence and interference of noise, diverse crack shapes, and blurred crack edges. Firstly, since the number of cracks in the borehole peeping results is usually relatively small, and the unbalanced sample distribution will affect the modeling effect. In real working conditions, there are extremely small and extremely large differences in the crack scales of the borehole peeping results, which requires the model to effectively extract multi-scale crack features. In addition, during the drilling process, the various interferences are often present, such as noise, lighting, image distortion, etc., which make the identification of cracks rather difficult. Moreover, the shape of the cracks is hard to predict, which may be linear, band-like or have complex geometric shapes. This requires the model to be capable of accurately identifying cracks of different shapes. Due to the fact that the transition between cracks and rocks in borehole imaging is usually rather ambiguous, boundary detection become even more difficult.

The crack data set collected from the 3303 working face of Hexi Coal Mine was analyzed through the ZKXG30 mine borehole peeping instrument. After intelligent recognition by the YOLOv8 model introducing the ResCBAM attention mechanism, the cracks in the surrounding rock of the roadway can be visually analyzed. By extracting cracks from the borehole peeping data set and combining multiple verification methods, the accuracy and reliability of model recognition can be improved. Given the substantial number of images requiring identification, it is impractical to present them all. Consequently, a selection of representative borehole observations has been chosen for display. These images reveal cracks exhibiting varying orientations and differing degrees of openness between them, illustrating the complexity of the subsurface structure. No significant fill material is discernible within these cracks, and the borehole walls demonstrate good integrity. Typical crack identification results are shown in Fig 7, where each pixel has undergone precise classification by the model and been labelled as either a crack or a non-crack. As shown in Fig 7, the model can identify crack cracks that are connected in different directions. If identified as a crack section, it shall be marked with a red bordered box, the pixels not constituting cracks shall remain unaltered, thereby clearly distinguishing them from the cracks. The model simultaneously outputs the accuracy of detected cracks. From the visual comparison results, the majority of crack identification and localisation outcomes appear satisfactory. The background interference caused by cutting operations around drill holes may affect crack identification results, leading to calibration of the red marker box being slightly larger than the actual crack location. Overall, under real mining conditions, the model accurately identifies and locates internal cracks visible through drill holes.

**Fig 7:**
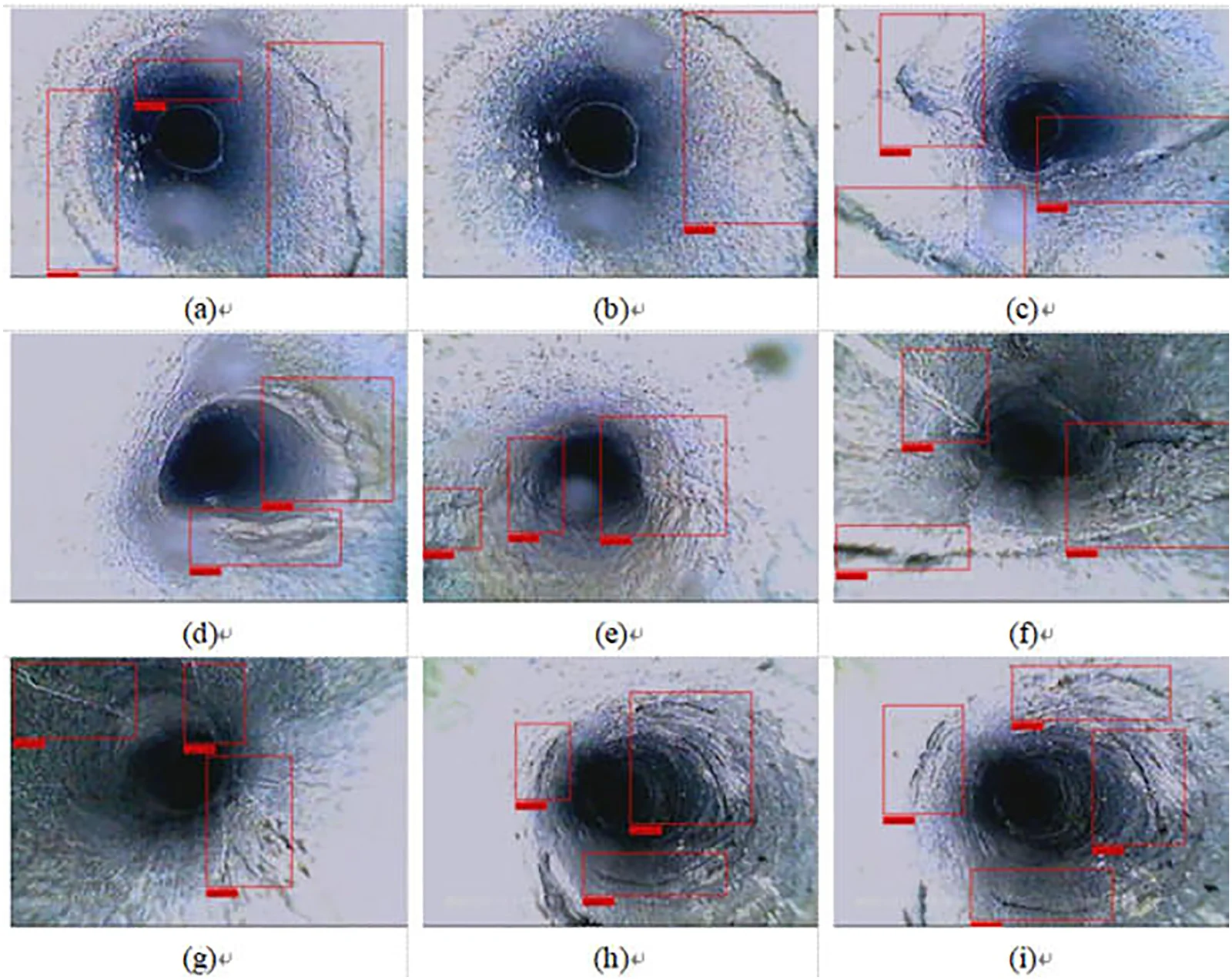
Identification results of cracks in some surrounding rock boreholes under the ResYOLOv8 model.

## 5. Evaluation and comparison of crack identification models

### 5.1 Model evaluation index

The borehole peeping crack recognition is equivalent to a pixel classification problem, that is, determining whether each pixel belongs to the background or the crack. The confusion matrix [28], as one of the commonly used evaluation indicators in classification problems, is often employed to measure the accuracy and performance of classification models during the prediction process. The confusion matrix is based on four different classification results, including True Positive (TP), True Negative (TN), False positive (FP), and False Negative (FN). Based on the confusion matrix, the commonly used evaluation metrics for image segmentation can be calculated, namely Precision, Recall and Mean Pixel Accuracy (MPA). Therefore, in the process of model training and performance verification, train loss, Precision, Recall, and MPA and the confusion matrix are adopted to quantitatively assess the model performance.

Among them, train loss is a core quantitative metric for measuring the fitting effect of the model on the training data set, which is used to characterize the degree of difference between the predicted output of the model and the real label. The magnitude of its value is positively correlated with the prediction error. That is, the smaller the train loss, the smaller the deviation between the prediction result of the model on the training set and the real label, and the higher the fitting degree of the model to the training data. Conversely, the greater the train loss, the greater the deviation between the predicted values of the model and the true values, and the worse the fitting effect. The cross-entropy loss function is adopted to identify the crack region and the non-crack region, and the calculation equation is:


$$\begin{array}{c} L=-\frac{\text{1}}{n}\sum_{i=\text{1}}^{n}\left[x_{i}log\left(a_{i}\right)+\left(\text{1}-x_{i}\right)log\left(\text{1}-a_{i}\right)\right] \end{array}$$ (5)

where *n* represents the number of pixels in the image, $x_{i}$ is the true label of the *i*-th pixel, the non-crack region is 0, the crack region is 1, and $a_{i}$ is the probability value of the *i*-th pixel predicting the target, which falls within the interval [0,1].

The Precision is a key indicator for evaluating the accuracy of a model’s positive sample prediction. It represents the proportion of samples correctly predicted as positive classes among all samples predicted as positive classes, reflecting the accuracy of the model’s recognition results. The calculation equation is:


$$\begin{array}{c} \text{Precision}=\frac{p_{ii}}{p_{ii}+p_{ji}} \end{array}$$ (6)

where $p_{ii}$ represents TP, which indicates the number of pixels with correct crack identification, and $p_{ji}$ represents FP, which indicates the number of pixels belonging to category *j* but identified as category *i*, that is, the number of pixels that are not crack but identified as crack. In the crack detection task of borehole peeping, the accuracy rate can be used to determine how many of the model’s predictions are accurate. Through the accuracy rate results, the true cracks can be obtained. The higher the precision rate, the lower the misjudgment rate of the positive sample prediction of the model and the higher the prediction accuracy. Conversely, the lower the precision rate, the more likely the model is to misjudge negative samples as positive ones.

The Recall is a core metric for evaluating a model’s ability to detect positive samples, representing the proportion of samples correctly predicted as positive to all actual positive samples, and reflecting the model’s coverage ability for crack targets. The calculation equation is:


$$\begin{array}{c} \text{Recall}=\frac{p_{ii}}{p_{ii}+p_{ij}} \end{array}$$ (7)

where $p_{ij}$ represents FN, indicating the number of pixels belonging to category *i* that are identified as category *j*, that is, the number of pixels whose cracks are identified as non-cracks. In the task of borehole peeping crack identification, the higher the recall rate, the stronger the model’s coverage ability for positive samples and the lower the missed detection rate, that is, the proportion of all truly existing positive samples that are successfully identified and predicted as positive by the model. The recall rate can be used to determine how capable the model is of finding all the actual existing cracks.

The MPA represents the proportion of correctly classified pixels to the total number of pixels. For the task of borehole peeping crack detection, the classification performance of the model at the pixel level can be known from MPA. The calculation equation is:


$$\begin{array}{c} \text{MPA}=\frac{\text{1}}{k+\text{1}}\sum_{i=\text{0}}^{k}\frac{p_{ii}}{\sum_{j=\text{0}}^{k}p_{ij}} \end{array}$$ (8)

where *k* represents the number of categories for image segmentation. The higher the MPA value, the higher the detection accuracy of the model for all types of targets and the lower the missed detection rate. Conversely, it indicates that the multi-category object detection performance of the model is worse.

### 5.2 Comparative experiments with different attention mechanisms

The attention mechanism can effectively enhance the model’s ability to identify cracks and effectively solve the problem that the color of cracks is similar to the background color and difficult to distinguish in the task of borehole peeping fracture detection. In order to find the optimal borehole peeping cracks detection model, this study adds two typical attention mechanisms, the SE and CBAM, respectively to the YOLOv8 algorithm for comparison with the ResCBAM attention mechanism. To ensure the rigor of the comparative experiments, all attention mechanisms are embedded in the last C2f module of the YOLOv8 feature extraction network, and the embedding positions are exactly the same as the operation procedures. Only the internal structure design of the attention mechanisms is changed to achieve single-variable control, ensuring that the differences in experimental results can be fully attributed to the structural characteristics of the attention mechanisms. The crack segmentation performance of different attention mechanism models on the training set and test set was quantitatively evaluated by using the evaluation metrics of train loss, Precision, Recall, MPA, and confusion matrix.

#### 5.2.1 SE-YOLOv8.

The core idea of SE attention mechanism is to enhance the performance of the model by compressing and stimulating the input features. Specifically, the SE attention mechanism consists of two steps: squeeze and excitation. In the squeeze step, the input feature map is compressed into a vector through a global average pooling operation, and then mapped to a smaller vector through a fully connected layer. In the excitation step, a sigmoid function is used to compress each element in this vector to between 0 and 1, and then it is multiplied by the original input feature map to obtain the weighted feature map. Through the SE attention mechanism, the model can adaptively learn the importance of each channel, thereby enhancing the model’s performance ability. In the SE-YOLOv8 model, the SE module is embedded in the feature output terminal of the C2f module. The overall structure is simple and lightweight. The attention mechanism is only implemented through pooling and fully connected layers. The increase in the number of parameters and computational load is small, and the impact on the model’s inference speed is weak. And it can effectively enhance the key semantic features of crack targets and improve the recognition sensitivity of small-scale cracks. The identification results of some surrounding rock borehole fractures through the SE-YOLOv8 model are shown in Fig 8. However, there is also the defect of ignoring the feature correlation of spatial dimensions, and the ability to locate cracks and capture edge contours is limited. Secondly, the global average pooling operation is prone to losing local detail information, and the robustness of feature calibration in complex backgrounds under borehole peeping is insufficient.

**Fig 8:**
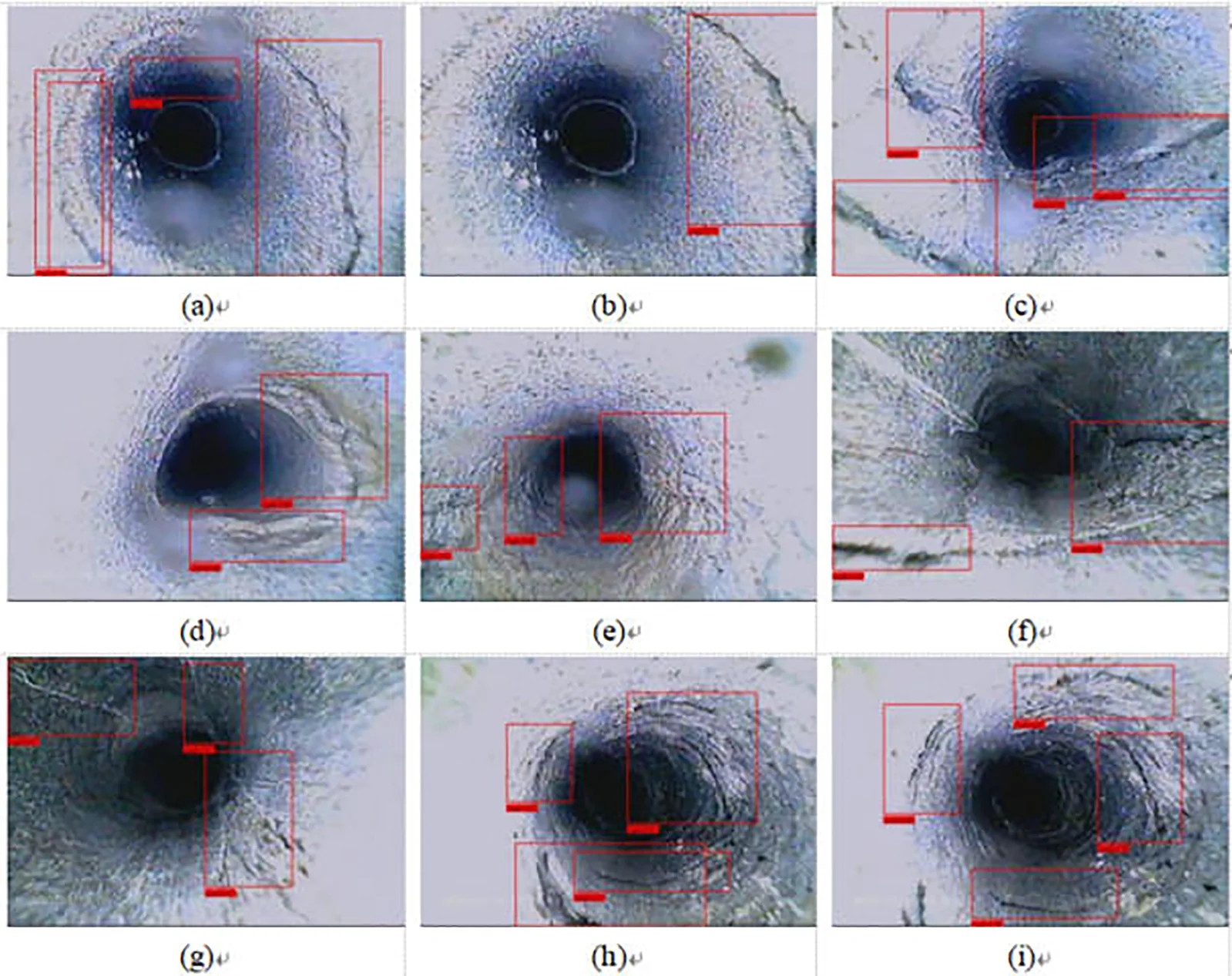
The identification results of cracks in some surrounding rock boreholes under the SE-YOLOv8 model.

#### 5.2.2 CBAM-YOLOv8.

The CBAM attention mechanism is a dual-branch attention module that integrates channel attention and spatial attention, and expands the attention dimension compared with the SE mechanism. The CBAM is used to adaptively adjust the key areas of focus in the image. The CBAM attention mechanism consists of two modules: the channel attention module and the spatial attention module. The channel attention module is used to learn the importance of each channel, enabling the network to focus more on those meaningful features while eliminating those meaningless ones. The input of this module is the output of a convolutional layer, and the output is the feature map after weight normalization. Specifically, for each channel, the channel attention module uses a global average pooling operation to obtain a feature vector, and then learns the importance of the channel through two fully connected layers and weights the final convolutional layer output. The spatial attention module is used to learn the importance of different spatial regions of an image, that is, the attention weights at different positions. The input of this module is the output of the convolutional layer, and the output is the attention matrix of each spatial position generated through convolution operations and fully connected operations in the spatial dimension. This matrix can adaptively adjust the size of the receptive field and focus on the key areas in the image. The channel and spatial attention modules can be combined to construct the CBAM attention mechanism. The application of this attention mechanism can improve the accuracy of tasks such as image classification, object detection and semantic segmentation. In the CBAM-YOLOv8 model, the CBAM module is also embedded in the feature output terminal of the last C2f module, and the channel attention and spatial attention calculations are performed successively in a serial structure. Here, the attention mechanism for both channel and spatial dimensions is simultaneously modeled, which not only strengthens the key feature channels but also precisely locates the spatial position of crack targets, enhancing the target discrimination ability in complex backgrounds. Secondly, the parallel design of pooling operations enhances the diversity of feature information and has a more comprehensive feature calibration capability compared to the SE mechanism. The identification results of some surrounding rock borehole fractures through the CBAM-YOLOv8 model are shown in Fig 9.

**Fig 9:**
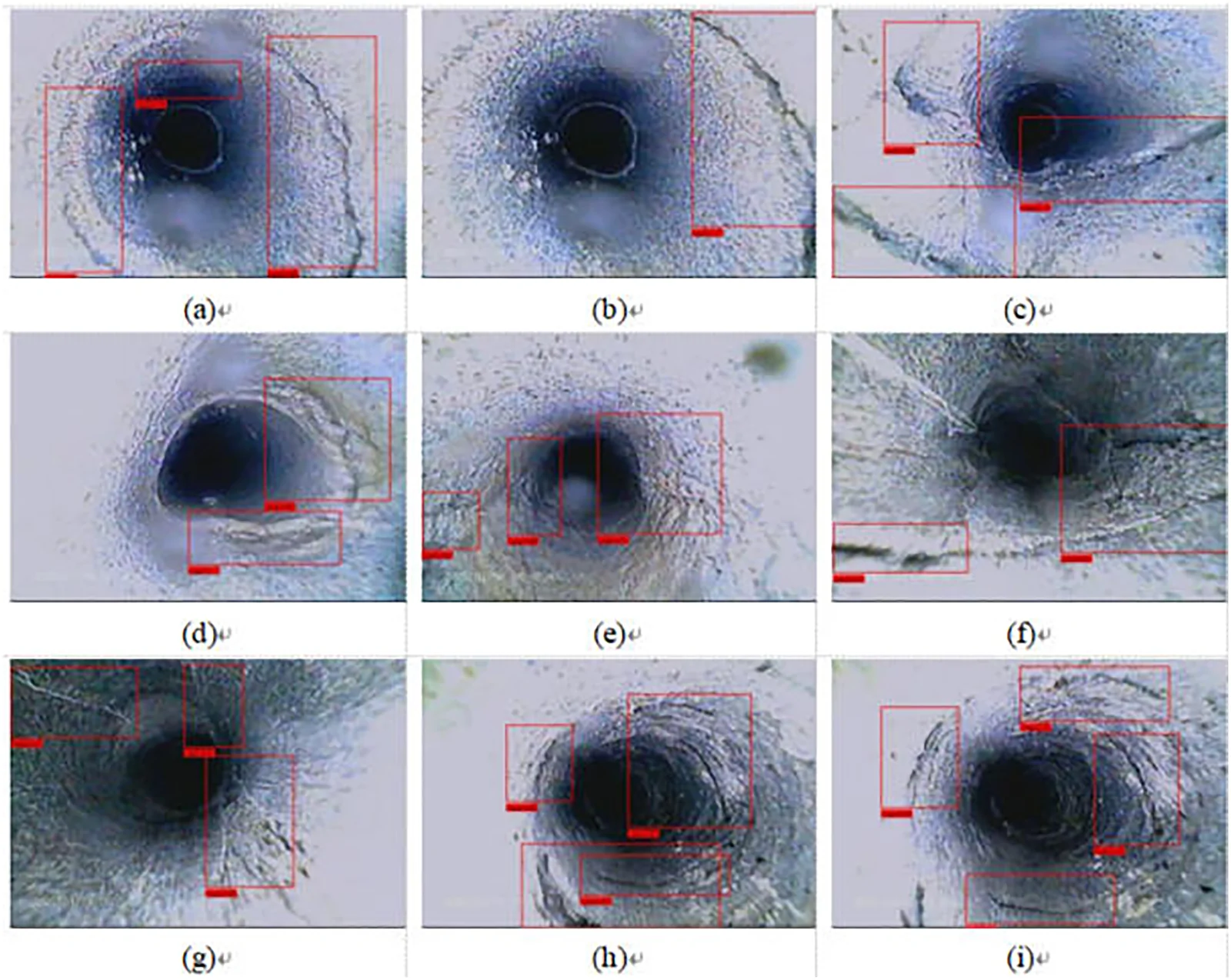
The identification results of fractures in some surrounding rock boreholes in the CBAM-YOLOv8 model.

### 5.3 Model performance verification and statistical analysis

#### 5.3.1 Ablation experiment.

Based on the YOLOv8 model, the residual ResBlock is introduced. When the number of network layers increases, the gradient attenuation is prone to occur, and the shallow crack feature is difficult to be updated. The residual connection realizes cross-layer information flow through short-circuit direct connection of branches, allowing gradients to be directly back-propagated, thereby improving the convergence ability of network training. Introducing the CBAM attention module can assign distinct weights to each feature channel. It enhances the weights of crack‑related feature channels while suppressing the weights of irrelevant channels induced by coal dust and stains, so as to improve the overall feature representation capability of the network. To verify the effectiveness of each improvement module, ablation experiments were conducted on the constructed dataset, and the model performance was analyzed based on three indicators such as Precision, Recall, and mAP50. The experimental results are shown in Table 1.

**Table 1:** Performance Comparison of Different Modules.

Model	Precision	Recall	mAP50
YOLOv8	83.86%	83.74%	89.53%
YOLOv8 + Res	86.58%	87.25%	92.20%
YOLOv8 + CBAM	90.96%	87.36%	92.45%
YOLOv8 + ResCBAM	92.96%	91.83%	93.77%

The results of the ablation experiments are shown in Table 1, which are used to verify the impact of the Res module, the CBAM attention module, and the ResCBAM combined module on the performance of identifying borehole fractures. To compare with the original YOLOv8 baseline model, after introducing the CBAM attention module alone, the accuracy rate increased by 7.10%, the recall rate increased by 3.62%, and the mAP50 increased by 2.92%, indicating that the CBAM attention can re-calibrate the features from the channel and spatial dimensions, strengthen the feature expression of the fracture target, suppress the interference of coal dust and surrounding rock background, and effectively improve the accuracy of fracture identification. When only the Res residual module is added, the accuracy rate increased by 2.72%, the recall rate increased by 3.51%, and the mAP50 increased by 2.67%. The residual structure optimizes the gradient propagation through short-circuit connections and retains the fine fracture features in the image, which can also improve the model’s recognition effect. When the residual structure is combined with the CBAM attention to form the ResCBAM module, the comprehensive performance of the model is further improved. Compared with the original YOLOv8 baseline model, the accuracy rate, recall rate, and mAP50 increased by 9.10%, 8.09%, and 4.24%, respectively. Compared with using CBAM alone or Res module alone, all indicators have improved. The above results show that the residual structure and the CBAM attention mechanism have a synergistic effect. The residual connection compensates for the loss of weak fracture features during the attention operation, and CBAM completes the weighted enhancement of the target features. The combination of the two can better adapt to the underground borehole endoscopic images and improve the model’s recognition performance of fracture targets.

#### 5.3.2 Statistical significance analysis.

To eliminate the experimental randomness caused by random weight initialization, all the ablation experiment groups maintained the same hyperparameters, datasets, and preprocessing. Each group of models was independently trained 5 times. The mean and standard deviation of each evaluation index are calculated according to equations (9) and (10), and the calculation results are shown in Table 2.

**Table 2:** Comparison of Mean and Standard Deviation of Detection Performance in Ablation Experiment Models.

Model	Average Precision/%	Average Recall/%	Average mAP50/%
YOLOv8	83.75 ± 0.17	83.74 ± 0.33	89.45 ± 0.35
YOLOv8 + Res	86.33 ± 0.26	87.61 ± 0.21	92.47 ± 0.27
YOLOv8 + CBAM	90.70 ± 0.27	87.46 ± 0.23	92.40 ± 0.10
YOLOv8 + ResCBAM	92.89 ± 0.08	91.25 ± 0.49	93.44 ± 0.30


$$\begin{array}{c} y=\frac{\text{1}}{m}\sum_{i=\text{1}}^{m}y_{i} \end{array}$$ (9)

where *y* represents the mean value, *m* is the number of repeated experiments, and *y*_*i*_ is the output value of the *i*-th experiment.


$$\begin{array}{c} S=\sqrt{\frac{\text{1}}{m-\text{1}}\sum_{i=\text{1}}^{m}{\left(y_{i}-y\right)}^{\text{2}}} \end{array}$$ (10)

where *S* represents the standard deviation.

From the standard deviations of each indicator in Table 2, it can be seen that the results of multiple training sessions for each group of models fluctuated little, indicating that the models have good stability. Further, an independent sample test was conducted on the mAP50 indicator between the ResCBAM-YOLOv8 model and the baseline YOLOv8 model.


$$\begin{array}{c} t=\frac{\overset{―}{y_{\text{1}}}-\overset{―}{y_{\text{2}}}}{\sqrt{\frac{{s_{\text{1}}}^{\text{2}}}{b_{\text{1}}}+\frac{{s_{\text{2}}}^{\text{2}}}{b_{\text{2}}}}} \end{array}$$ (11)

where the *y*_1_ represents the mean mAP50 value of the baseline YOLOv8, *y*_2_ represents the mean mAP50 value of ResCBAM-YOLOv8, *s*_1_ represents the sample standard deviation of the baseline, *s*_2_ represents the sample standard deviation of ResCBAM, *b*_1_ and *b*_2_ represent the number of repeated training times, and *b*_1_ = *b*_2_ = 5.

According to Eq. (11), it is calculated that p = 7.44 × 10^−8^, which is much smaller than 0.05. This proves that the improvement in detection performance brought by the ResCBAM module is statistically significant, and this improvement is not an accidental result caused by random initialization.

### 5.4 Comparison results

The evaluation index results of the borehole peeping crack recognition model established by combining multiple attention mechanisms with YOLOv8 are shown in Fig 10. As can be seen from the Fig 10, with the increase of the number of iterations, the precision rate, recall rate and MPA all gradually increase. When the number of iterations reaches more than 100 times, all three are basically stable, and the MPA is greater than 85% and gradually increases, indicating that the model has a strong crack recognition ability and high recognition accuracy.

**Fig 10:**
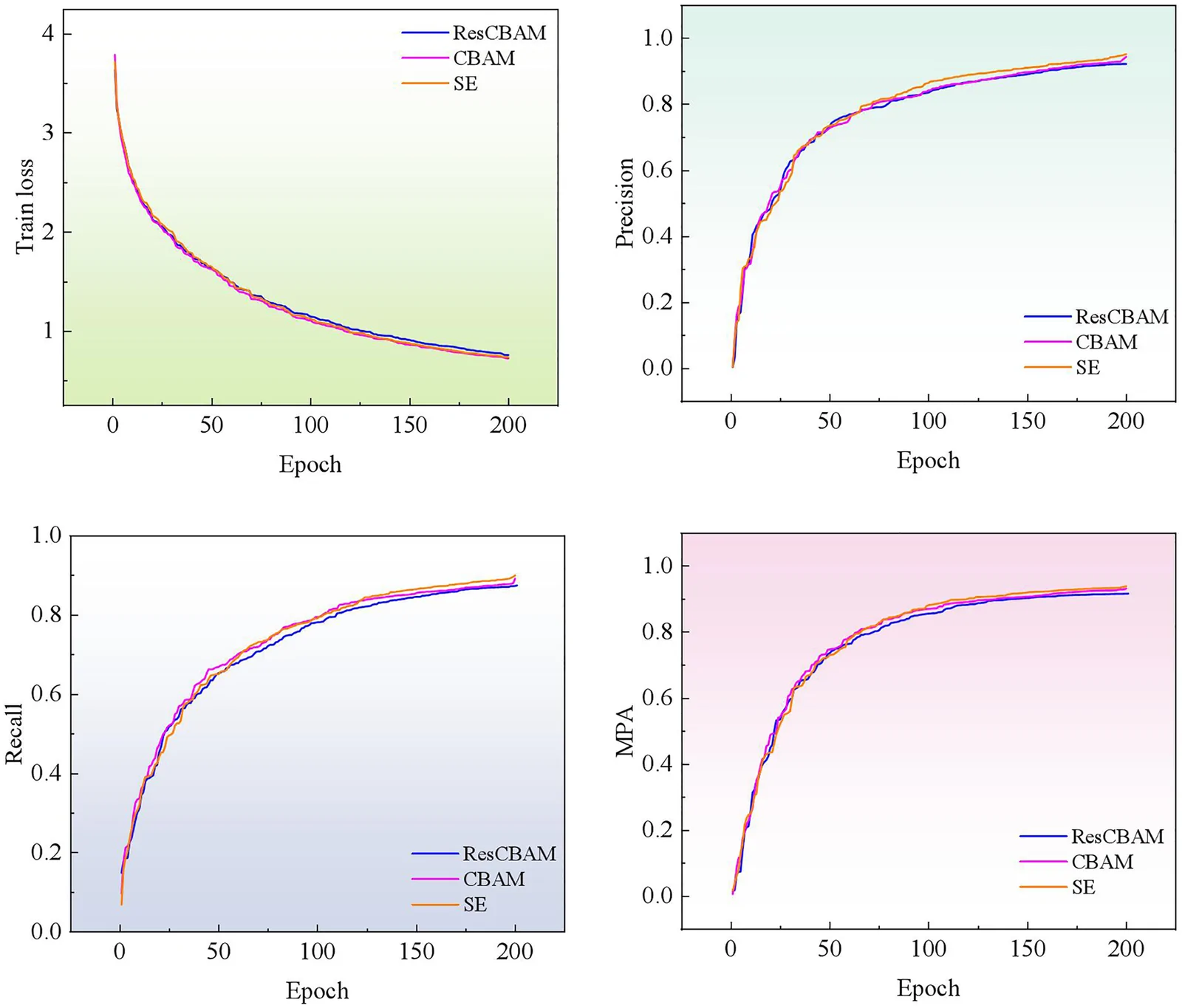
Comparison of different models in comparison experiments.

The standardized confusion matrix of the crack identification model is shown in Fig 11. Each row of this matrix corresponds to the true category of the data, and each column corresponds to the predicted category of the model. Therefore, each element in the matrix represents the matching situation between the true category and the predicted category. The two-dimensional matrix presents the prediction results of the model for crack identification, including four dimensions: true positive, true negative, false positive and false negative. It can be seen from the confusion matrix that the ResCBAM-YOLOv8 model accurately identified 92% of the fractures inside the boreholes of the mine working face, and only 8% were misjudged as false negatives. In comparison, the SE-YOLOv8 model accurately identified 91% of the fractures inside the boreholes in the mine face, while 9% were misjudged as false negatives. The CBAM-YOLOv8 model accurately identified 90% of the fractures inside the boreholes in the mine working face, while 10% were misjudged as false negatives. Therefore, compared with the identification model [29–32], the ResCBAM attention mechanism composed of the channel attention module, the spatial attention module and the residual connection can better enhance the model’s ability to identify fractures inside the mine.

**Fig 11:**
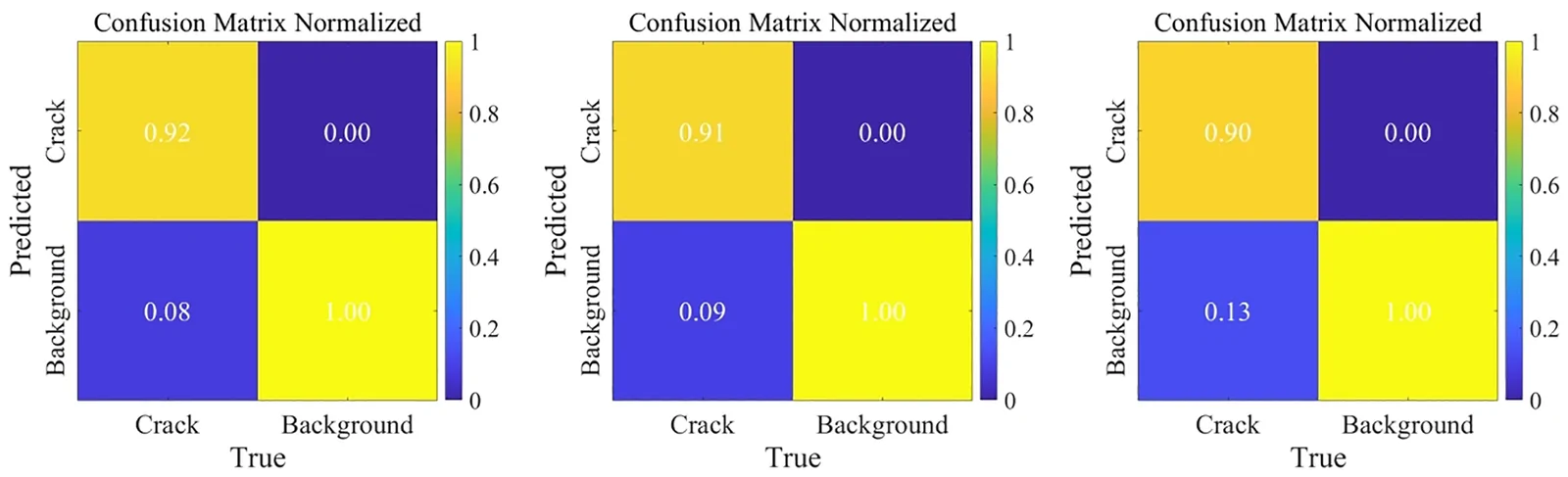
Standard confusion matrix.

## 6. Conclusions

This study reaches the following conclusions:

(1) A ResCBAM-YOLOv8 network model suitable for the detection of internal fractures in borehole peeping was proposed, which achieved the accurate detection of internal fractures in real mine working faces. A borehole peeping fracture image data set was fabricated, and the fracture data set was manually labeled using Labelme.
(2) The original fracture images are optimized through preprocessing methods such as brightness enhancement, contrast adjustment, equalization, denoising, and filtering on the borehole peeping fracture data set, facilitating subsequent modeling for fracture identification. The constructed ResCBAM-YOLOv8 network model can also achieve ideal detection effects under complex fissure conditions.
(3) To verify the feasibility of the constructed ResCBAM attention mechanism in the YOLOv8 crack recognition model, typical attention mechanisms SE and CBAM were respectively added to the YOLOv8 algorithm for crack recognition. By comparing the results of the confusion matrix with those of training loss, precision, recall and MPA, it can be known that ResCBAM-YOLOv8 has achieved better performance in the detection of fractures in real mines, especially showing significant improvement in the detection of edge and small target fractures.

This work mainly conducts ablation comparison experiments for different attention modules within the CNN framework. However, a complete comparison experiment with advanced Transformer-based crack detection models such as Swin-Transformer has not yet been carried out. Transformer-based models possess strong global modeling capabilities, but they have higher parameter quantities and computational costs. In the subsequent research, a unified training configuration will be adopted. On the drilling fracture dataset, the Transformer architecture and the dedicated crack detection network will be trained and tested to further comprehensively evaluate the fracture recognition performance and engineering applicability of the ResCBAM-YOLOv8 model. The development of fractures affects the deformation and stability of the rock mass. Changes in the rock mass structure can lead to geological disasters. It is recommended that the mining department pay close attention to monitoring and predicting the development of internal cracks in boreholes, regularly acquiring information on fracture evolution along the borehole walls using borehole imaging devices, and tracking crack propagation patterns under mining-induced disturbances. The development status of borehole fractures should be incorporated into the surrounding rock risk early-warning system, with increased monitoring density in high-risk areas, thereby guiding support design and excavation operations to reduce the risk of dynamic disasters triggered by surrounding rock instability. The development of fractures affects the deformation and stability of the rock mass. Changes in the rock mass structure can lead to geological disasters. It is recommended that the mining department pay close attention to monitoring and predicting the development of internal cracks in boreholes, regularly acquiring information on fracture evolution along the borehole walls using borehole imaging devices, and tracking crack propagation patterns under mining-induced disturbances. The development status of borehole fractures should be incorporated into the surrounding rock risk early-warning system, with increased monitoring density in high-risk areas, thereby guiding support design and excavation operations to reduce the risk of dynamic disasters triggered by surrounding rock instability. The development characteristics of rock fractures have a significant impact on the mechanical properties of the surrounding rock and the stability of the roof. The evaluation of roof stability is a comprehensive geotechnical engineering analysis issue. Besides the information on fracture development, multiple mechanical and engineering parameters such as the strength of the surrounding rock, ground stress, and support conditions need to be coupled to establish a quantitative relationship between the fracture characteristics and roof instability. Subsequent research will be based on the fracture identification results obtained in this work, and further conduct correlation analysis between fracture characteristics and roof stability, with the aim of providing more complete technical means for roof risk identification.
